# 

**Published:** 1900-07-28

**Authors:** 


					CADBURY'S U CADBURY'S
MM Jg* cocoa _
NO DRUGS OR ALKALIES USED. ^
COCOA
absolutely pure
r
No. 721 Vol. XXVIII. CSSSta Saturday,
July 28, 1900.
[SubBCriptkra TeraiB,] pRICE TWOPENCE.

				

